# Quantification of the Aluminum Content Leached into Foods Baked Using Aluminum Foil

**DOI:** 10.3390/ijerph17228357

**Published:** 2020-11-12

**Authors:** Paola Fermo, Gabriele Soddu, Alessandro Miani, Valeria Comite

**Affiliations:** 1 Dipartimento di Chimica, Università degli Studi di Milano, 20133 Milano, Italy; gabrielesoddu95@gmail.com (G.S.); valeria.comite@unimi.it (V.C.); 2 Italian Society of Environmental Medicine (SIMA), 20133 Milano, Italy; alessandro.miani@gmail.com; 3 Department of Environmental Science and Policy, University of Milan, 20133 Milano, Italy

**Keywords:** aluminum, leaching, ICP-OES, metals migration, SEM-EDS

## Abstract

In this study, determinations of the aluminum content in meat and fish performed after having cooked these foods using commercially available aluminum foil have been performed. The release of this chemical element was evaluated by cooking beef, chicken, and fish wrapped with commercial aluminum foil using seasoning or without seasoning in order to evaluate the effect on Al leaching into the food. The characterization of the food samples was carried out using two different analytical methods: inductively coupled plasma optical emission spectrometry (ICP-OES), for the quantification of aluminum, and scanning electron microscopy coupled with energy dispersion spectroscopy (SEM-EDS) to evaluate any structural changes occurring inside the aluminum foil after the cooking procedure. It has been demonstrated that the leaching that occurs when the foods are cooked by wrapping them in Al foil is not negligible and that the consumption of these foods, together with the consumption of other foodstuffs, such as, for example, some vegetables that may naturally contain aluminum, can lead to consuming a weekly dose not far from the TWI (tolerable weekly intake).

## 1. Introduction

Food chemical analysis is a unique tool for the evaluation of both the quality and safety of foods, and nowadays there is an increasing need to have accurate knowledge of food chemical composition [1]. This great interest is evidenced by a constantly increasing number of publications on this topic in the more recent scientific literature. Most of these publications are focused on the determination of both chemicals and bio-markers in order to assess toxicity to humans or obtain information on environmental pollution [2,3,4].

It is well known that aluminum (Al) is a widespread natural element (the third most common element in the Earth’s crust [5]) and as a consequence is contained in different kinds of matrices, such as water, soil, and food [1,4,6,7,8]. Additionally, aluminum is widely employed in many industrial activities and present in products such as cosmetics, deodorants (which typically contain aluminum salts), or body care creams. Furthermore, it is widely employed as aluminum foil used for food preparation, cooking, and also for foodstuff storage and packaging [9,10]. However, over the last decades, the toxicity of aluminum to humans has been a topic of discussion and is still not completely clarified. The neurotoxicity of Al has been proven [11], and it has also been found that chronic aluminum intake causes Alzheimer’s disease [12].

Dietary intake is the main source of exposure to aluminum for humans. From the available data, it is known that cereals and products derived from them, vegetables, and beverages, are the main vectors of exposure.

Aluminum is widely used in the food sector for the production of packaging and containers that are in contact with food, and it is recognized that the release of Al from the packaging into the foodstuff represents a risk [4,5,9]. In the event that aluminum comes into contact with particularly aggressive drinks or foods (i.e., those capable of favoring Al leaching), such as those high in acidity or with high salt content, in order to prevent leaching the containers are internally coated with a layer of polymer that insulates the aluminum from direct contact with food [13].

Another concern is represented by aluminum utensils, which are ubiquitous in households of developing countries. In particular, aluminum shows pathological effects on the human body (such as anemia, dementia, and osteomalacia) due to its leaching from utensils with long-term usage.

The use of aluminum tools and foils therefore represents another relevant source of aluminum that contributes to increasing the quantities of aluminum consumed through food [14].

Numerous studies have recently focused on the assessment and quantification of aluminum release [11,15,16,17]. Al has low bioavailability in healthy humans, but the absorbed dose has a certain capacity for bioaccumulation.

An in-depth study was carried out by the European Food Safety Authority (EFSA), which drafted a detailed report on the safety of aluminum from dietary intake [18]. The World Health Organization (WHO) and EFSA have established a tolerable weekly intake (TWI) for a human subject of 1 mg of aluminum per kg of body weight per week (i.e., 1 mg/kg bw/w) [17,18,19].

For example, the estimated daily intake of Al was calculated for the Belgian adult population and amounted to 0.030 mg/kg body weight/day [20] or 21% of the provisional tolerable weekly intake (PTWI), which was established in 2008 and subsequently confirmed by the EFSA [19].

However, more generally, the limit of 1 mg/kg bw/w is often exceeded by a large part of the population: on average, an adult consumes, through diet alone, a quantity between 0.2 and 2.5 mg/kg bw/w, while for a child the range oscillates between 0.7 and 2.3 mg/kg bw/w [16].

In Italy, Istituto Superiore di Sanità also published an in-depth study on the release of Al from the contact materials in 2008 [13] while the Italian Ministry of Health published an opinion paper in 2017 [16].

It is difficult to establish what effects the long-term intake of limited quantities (chronic toxicity) may have, such as that which occurs due to repeated ingestion of small quantities of Al through the diet. For this reason, extensive research has also been carried out on the different sources of aluminum in order to plan the reduction of the quantities ingested. Children, who generally eat more food than adults in relation to their weight, represent the category with the highest potential for exposure to aluminum per kg bw/w [18]. In fact, the potential exposures generally come from foods and specific preparations for infants.

In 2013, the European Council (EC) approved limits for metals and alloys that can come into contact with food [4]: specific release limits (SRLs) from metals and alloys into food have been indicated in this regard, including for aluminum. The SRL for the release of aluminum to food has been specified not to exceed 5.00 mg/kg of food. According to EC regulation n. 1935/2004 [13], items (for packaging and wrapping) intended for contact with food must not release their constituents in quantities that could represent a danger to human health, lead to an unacceptable change in the composition of the food, or lead to a deterioration of the organoleptic characteristics.

In order to assess the exposure to aluminum for humans, numerous studies have been recently carried out, including the evaluation of Al leaching from aluminum cookware in meat and milk [21] and the evaluation of leaching from aluminum foil used for cooking food such as meat or fish [5,22,23,24,25] that are wrapped with aluminum foil and cooked in an oven.

The complexing effect plays an important role in the aluminum release process [14] within food. In fact, complexing ions that are present in the food itself (such as organic acids) or within the seasoning used (for example, salt or lemon juice) increase the released Al concentration. Furthermore, at pH values typical of most foods (i.e., between 4 and 8), aluminum is mainly present in the form of organic complexes that are harmful to humans. The release, in fact, consists of the migration of aluminum into the food in the form of Al^3+^ ions, which can subsequently be complexed.

The quantities released by the foils depend on factors such as pH, salinity, the fat content of the food, temperature, and exposure time. Low pH values favor an increase in release; therefore, aluminum migrates more easily when it is in contact with acidic foods, but also with products rich in salt, with a trend depending on the time and contact temperature [13,26]. For example, some studies [25] on foods wrapped in aluminum foil subjected to different cooking times have shown a correlation between parameters such as temperature, cooking time, and type of food and the Al concentrations found in the sample.

In the present study, the Al content in meat and fish wrapped in aluminum foil and cooked in an oven was quantified by elemental analysis by using inductively coupled plasma optical emission spectrometry (ICP-OES) with the aim of evaluating Al released into these kinds of foods. Furthermore, the appearance and morphological features of the foil after baking were evaluated by scanning electron microscopy coupled with energy dispersion spectroscopy (SEM-EDS), which showed the presence of microscopically small holes.

## 2. Materials and Methods

### 2.1. Samples Preparation

Chicken, beef, and fish samples (about 100 g of product) were baked in a commercial brand oven, such as those commonly found in domestic kitchens, for 1 h at a temperature of 180 °C. These conditions were chosen as they are considered representative of the typical household cooking conditions for this kind of food. For each type of food, three tests were carried out: a sample was cooked in a Pyrex pan, a sample was wrapped in aluminum foil, and a sample was wrapped in aluminum foil in the presence of seasoning (oil, salt, and lemon for chicken and fish, oil and salt for meat). The aluminum foil used was a commercial brand available on the market. The foil used had a thickness of about 20 μm and a chemical composition, tested by SEM-EDS analysis, that corresponded to 100% by weight of Al. All the prepared meat and fish samples are reported in Table 1 (acronyms are shown in the table).

Subsequently, after cooking, the samples were minced and a known quantity (about 1 g) was taken; the samples obtained were placed in Teflon containers to which a mixture of 12 mL 1:3 of HNO_3_: HCl was added. Heating was then carried out on a hot plate for 1 h, until the contents were almost completely dissolved. When dissolution was complete, the samples were left to stand until the following day at room temperature; then the solution was filtered with a 45-µm microfilter and brought to volume in a 50-mL flask with MilliQ water. Next, 5 mL solutions were taken from it, placed in 10-mL flasks and made up to volume with MilliQ water. Each sample was prepared in triplicate. After dilution, the samples were stored in a refrigerator.

### 2.2. Samples Analysis

Aluminum quantification was carried out by inductively coupled plasma optical emission spectrometry (ICP-OES) using an instrument model Optima 8000 (Perkin Elmer, Waltham, MA, USA). Standard aluminum solutions with concentrations in the range of 0.2–2 ppm were prepared for the construction of the calibration curve. A QA/QC (quality assurance/quality control) procedure was followed, and it was verified that the standard deviation calculated on the three samples for each batch was lower than 10% in all the cases; Al recovery was verified by spiking blank with a standard of known concentration.

For chicken and beef, three samples of aluminum foil used for cooking were analyzed by a Hitachi TM1000 Tabletop Scanning Electron Microscope (SEM) coupled with an Oxford EDS (energy dispersion spectroscopy) unit (Hitachi Italia, Brugherio, Italy). One sample was taken from a point in contact with the meat cooked without any seasoning (sample Al-C-BNS and Al-C-CNS in Table 2), one sample was taken from a point in contact with the meat cooked with seasoning (sample Al-C-BS and Al-C-CS in Table 2) and a third sample was taken from a point where the foil was not in direct contact with the flesh cooked with seasoning (sample Al-N-BS and Al-N-CS in Table 2).

To carry out the observations, the samples were mounted on the sample holder stub of the instrument using a double-sided adhesive graphite disc. EDS analyses [27] were performed by the Oxford EDS probe.

## 3. Results and Discussion

### 3.1. Determination of Al Content in Food

It is well known that wrapping meat or fish with aluminum foil for cooking in an oven is a common practice, which is also typical of Italian cuisine. In order to assess the aluminum quantity leached from the Al foil into the food after cooking, two kinds of meats (beef and chicken) and fish were chosen taking also into account that data on these foodstuff were partly available in the literature [5,13,22,23,24,25,26]. Furthermore, in the literature, disposable aluminum trays have also been tested for their capacity to release Al [13].

**Figure 1:**
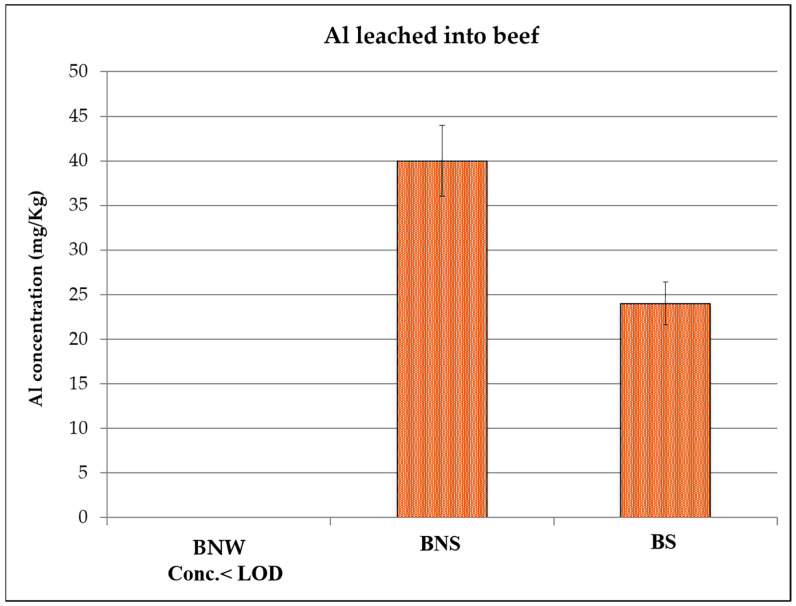
Aluminum concentration detected in beef baked without wrapping in aluminum foil (BNW), beef baked in aluminum foil without seasoning (BNS), and beef baked in aluminum foil with some seasoning (BS)**.** LOD: limit of detection.

**Figure 2:**
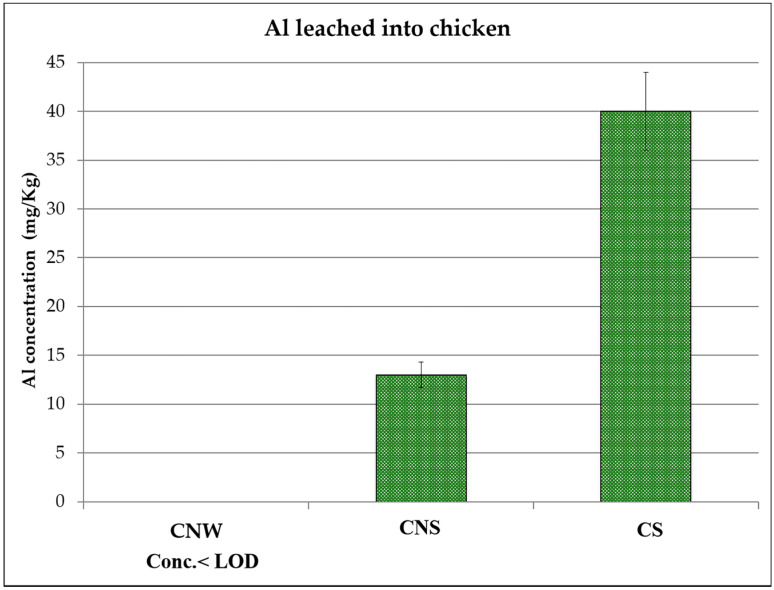
Aluminum concentration detected in chicken baked without wrapping in aluminum foil (CNW), chicken baked in aluminum foil without seasoning (CNS), and chicken baked in aluminum foil with some seasoning (CS). LOD: limit of detection.

**Figure 3:**
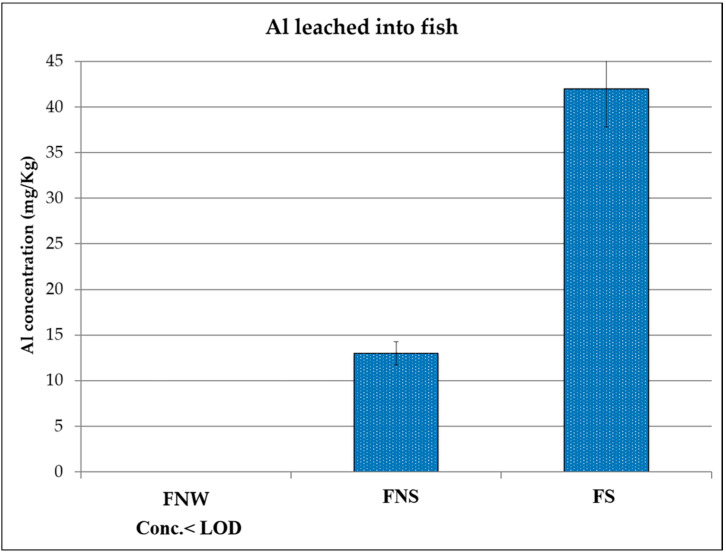
Aluminum concentration detected in fish baked without wrapping in aluminum foil (FNW), fish baked in aluminum foil without seasoning (FNS), and fish baked in aluminum foil with some seasoning (FS). LOD: limit of detection.

The Al concentrations determined for the three kinds of examined foodstuffs are reported in Figure 1, Figure 2 and Figure 3 (see also Table 1 for the acronyms used to identify the samples). In each figure, the following results are reported: the Al content in the specific food cooked in a Pyrex pan in an oven, the Al content in the same food wrapped in Al foil without adding any seasoning and cooked in an oven, and the Al content in the same food, but with seasoning added, wrapped in Al foil and cooked in the same oven.

For all three samples, it was found that when cooking without the foil (i.e. using the Pyrex pan), Al concentration was below the limit of detection of the technique (values reported as < LOD (limit of detection) in Figure 1, Figure 2 and Figure 3). The same was observed for fresh meats and the fish, i.e. not cooked, and analyzed as reference blanks.

For fish and chicken (Figure 2 and Figure 3), the maximum concentrations (42 mg/Kg for fish and 40 mg/Kg for chicken) were detected for the samples that were wrapped in Al foil and cooked with seasoning, while in the case of beef (Figure 1), the maximum value (40 mg/Kg) was found in the sample wrapped in Al foil but cooked without adding seasoning. This result could be due to the fact that beef has a high fat content: from the literature, it is known that Al leaching depends on different parameters [14], including not only temperature but also pH, salinity, and food composition, and in the case of beef, Al could be linked to some organic acids present in the meat, resulting in a high uptake from the foil. More replicates and further tests, also including tests on different kinds of beef, would be necessary to better explain this phenomenon. What can be observed in general is that for all the analyzed samples coming from food wrapped in foil, the release of aluminum occurred in non-negligible quantities. In fact, taking into account the maximum exposure limits suggested by the EFSA [18], the intake of these foods several times a week, together with the intake of aluminum from other sources that naturally contain Al (such as vegetables), could lead to reaching the TWI (tolerable weekly intake).

Furthermore, the values determined in the present study are in agreement with what has been reported in other studies [22,24,25], even if the cooking conditions were slightly different.

It is worth noting that the aluminum foil chosen in this study to wrap the foods is representative of the brands on the market. However, on the basis of some preliminary tests that will deserve further study, it has been observed that the concentration of metal released also depends on the foil characteristics (for example, the thickness). In particular, the foil employed in the present research is among the thinnest available on the market, but the use of a thicker foil, which corresponds to greater strength, could potentially lead to the release of more Al into the food.

### 3.2. Aluminum Foil Morphological Observation by SEM-EDS

**Figure 4:**
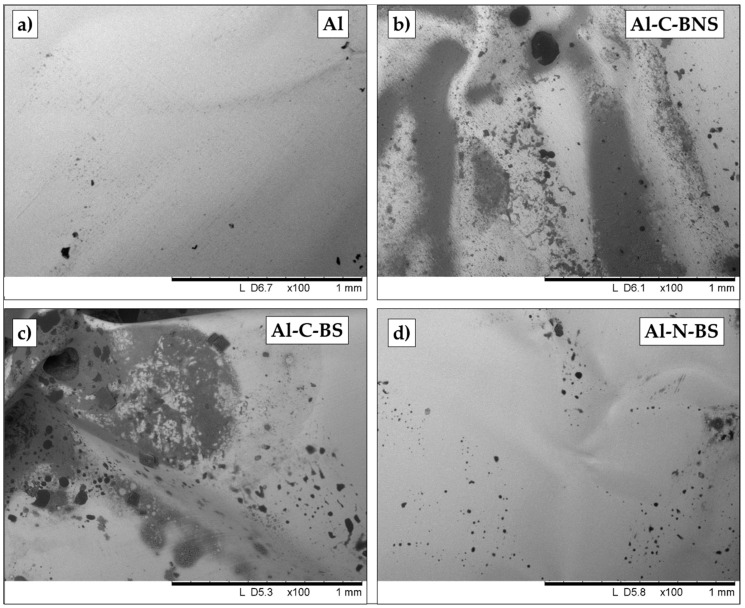
SEM images acquired from (**a**) aluminum foil in its unused state; (**b**) aluminum foil used for baking beef without seasoning and taken from a portion which was in contact with the beef (Al-C-BNS: Al contact beef, no seasoning); (**c**) aluminum foil used for baking beef with seasoning and taken from a portion which was in contact with the beef (Al-C-BS: Al contact beef, with seasoning); (**d**) aluminum foil used for baking beef with seasoning and taken from a portion which was not in contact with the beef (Al-N-BS: Al no contact beef, with seasoning).

**Figure 5:**
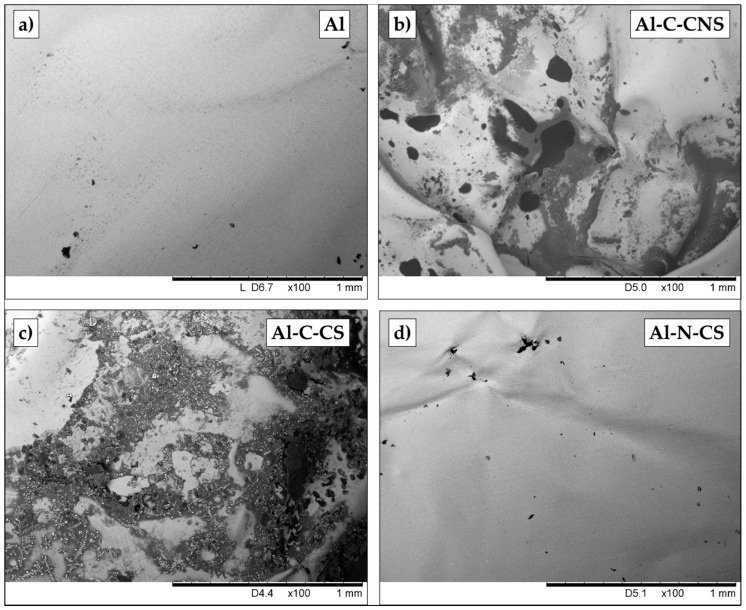
SEM images acquired on (**a**) aluminum foil in its unused state; (**b**) aluminum foil used for baking chicken without seasoning and taken from a portion which was in contact with the chicken (Al-C-CNS: Al contact chicken, no seasoning); (**c**) aluminum foil used for baking chicken with seasoning and taken from a portion which was in contact with the chicken (Al-C-CS: Al contact chicken, with seasoning); (**d**) aluminum foil used for baking chicken with seasonings and taken from a portion which was not in contact with the beef (Al-N-CS: Al no contact chicken, with seasoning).

In Figure 4 and Figure 5, the comparison among portions of Al foil employed for wrapping the different foods is reported (Figure 4 refers to beef and Figure 5 refers to chicken). As a comparison, in both the figures a portion of Al foil in its unused condition was also reported. It is possible to observe a marked deterioration of the surface of the foil that was in direct contact with the meat. In fact, the surface had numerous holes with variable diameters in the range of 100–150 μm for the samples cooked without seasoning (Figure 4b and Figure 5b) and slightly larger and more numerous holes for the samples where seasoning was added (Figure 4c and Figure 5c), although in the case of chicken, the effect of adding seasoning was mainly a higher deterioration process of the foil (Figure 5c). On the contrary, unused Al foil had no holes on the surface (Figure 4a and Figure 5a). It is also interesting to observe how in the case of the portion of Al foil that was not in direct contact with the meat, the number of holes is lower (Figure 4d and Figure 5d) and also the size of the holes is slightly lower. The presence of holes in the foils in contact with the food obviously confirms the migration of aluminum from the foil to the food.

Some morphological observations made by SEM on deteriorated foil after cooking have been previously reported in the literature [26]; nevertheless, to our knowledge, this is the first time that a systematic comparison among foils baked in different conditions (with or without seasoning for the same food) has been reported.

### 3.3. General Considerations

It is worth noting that the tests performed in the present research, even if preliminary (mainly due to the fact that a limited number of cases were examined and also because the study considers only one kind of Al foil), have been confirmed in a recent report published by the Italian Ministry of Health in 2019 [28], where it was stated that the results obtained also confirm the conclusions reported in a previous document from the same Ministry from 2017 [16].

Regarding Al migration, it should be emphasized that attention should be paid to the potential health risk posed to the most vulnerable groups, especially to children under 3 years of age.

According to the reports by the Italian Ministry of Health, results obtained in this research should arouse attention and concern, since the possible exposure to the consumer could lead to exceeding the TWI (tolerable weekly intake) as based on the value defined by the EFSA in 2008 [18].

In addition, the results of this study suggest how other kinds of materials (for example, baking paper) or techniques should be preferential used for baking foods.

## 4. Conclusions

The aim of this study was to demonstrate that under real cooking conditions, the phenomenon of aluminum leaching from aluminum foil to the food occurs and should arouse attention and concern.

On the basis of the results obtained (although preliminary and in need of further testing for example, by analyzing different types of meats exposed to different cooking temperatures and cooking times) the release of aluminum was demonstrated in foods that were cooked while wrapped in a commercial aluminum foil. The concentrations found were in line with those listed in previous studies reported in the scientific literature. The release of aluminum was also confirmed in samples cooked without seasoning.

Finally, in our opinion, it is important to underline the conclusion of the Italian Ministry of Health, who recommended that aluminum should be included as a priority in the MOCA (materials and objects in contact with food) monitoring plan in order to collect an adequate database at the national level and this study represents a contribution in this sense. In conclusion, on the basis of the results obtained from this study, is recommended that the use of Al foil for baking food should be avoided in order not to exceed the TWI suggested for Al by health authorities such as the Italian Ministry of Health.

## Figures and Tables

**Table 1:** Foods analyzed (beef, chicken, and fish): samples baked without wrapping (i.e. no wrapping, NW) with the aluminum foil (BNW, CNW, FNW, respectively); samples wrapped in the aluminum foil and baked without seasoning (BNS: beef, no seasoning; CNS: chicken, no seasoning; FNS: fish, no seasoning); samples wrapped in the aluminum foil and baked using some seasoning (BS: beef with seasoning; CS: chicken, with seasoning; FNS: fish, with seasoning).

Food Sample	Food Baked without Wrapping	Food Baked without Seasoning	Food Baked with Seasoning
Beef	BNW	BNS	BS
Chicken	CNW	CNS	CS
Fish	FNW	FNS	FS

**Table 2:** Aluminum samples taken from the wraps used for baking beef and chicken and analyzed by SEM: Al-C-BNS: Al contact beef, no seasoning; Al-C-BS: Al contact beef, with seasoning; Al-N-BS: Al no contact beef, with seasoning; Al-C-BNS: Al contact chicken, no seasoning; Al-C-BS: Al contact chicken, with seasoning; Al-N-BS: Al no contact chicken, with seasoning.

Beef	Chicken
Al-C-BNS	Al-C-CNS
Al-C-BS	Al-C-CS
Al-N-BS	Al-N-CS
